# Frequent Changes in Expression Profile and Accelerated Sequence Evolution of Duplicated Imprinted Genes in *Arabidopsis*

**DOI:** 10.1093/gbe/evu144

**Published:** 2014-07-02

**Authors:** Yichun Qiu, Shao-Lun Liu, Keith L. Adams

**Affiliations:** ^1^Department of Botany, University of British Columbia, Vancouver, British Columbia, Canada; ^2^Department of Life Science, Tunghai University, Taichung, Taiwan

**Keywords:** duplicated genes, whole-genome duplication, imprinted genes, neofunctionalization

## Abstract

Eukaryotic genomes have large numbers of duplicated genes that can evolve new functions or expression patterns by changes in coding and regulatory sequences, referred to as neofunctionalization. In flowering plants, some duplicated genes are imprinted in the endosperm, where only one allele is expressed depending on its parental origin. We found that 125 imprinted genes in *Arabidopsis* arose from gene duplication events during the evolution of the Brassicales. Analyses of 46 gene pairs duplicated by an ancient whole-genome duplication (alpha WGD) indicated that many imprinted genes show an accelerated rate of amino acid changes compared with their paralogs. Analyses of microarray expression data from 63 organ types and developmental stages indicated that many imprinted genes have expression patterns restricted to flowers and/or seeds in contrast to their broadly expressed paralogs. Assays of expression in orthologs from outgroup species revealed that some imprinted genes have acquired an organ-specific expression pattern restricted to flowers and/or seeds. The changes in expression pattern and the accelerated sequence evolution in the imprinted genes suggest that some of them may have undergone neofunctionalization. The imprinted genes *MPC*, *HOMEODOMAIN GLABROUS6 *(*HDG6*), and *HDG3* are particularly interesting cases that have different functions from their paralogs. This study indicates that a large number of imprinted genes in *Arabidopsis* are evolutionarily recent duplicates and that many of them show changes in expression profiles and accelerated sequence evolution. Acquisition of imprinting is a mode of duplicate gene divergence in plants that is more common than previously thought.

## Introduction

Ongoing gene duplication events during evolutionary history have provided new genes that can diverge in function and gain new functions, leading to new morphological and physiological characteristics. There are several different types of gene duplication events. The largest scale of gene duplication is whole-genome duplication (WGD), which doubles the entire genome. WGDs at various evolutionary time scales have been shown across eukaryotes including yeasts, animals, and plants (reviewed in Van de Peer et al. 2009). All angiosperms have experienced at least one round of ancient WGD event at the early stage of their evolutionary history, and many lineages have one or more additional polyploidy events (Cui et al. 2006; Soltis et al. 2009). In particular, the *Arabidopsis* lineage has experienced two WGD events after the divergence from a common ancestor with *Carica* (papaya) in the order Brassicales (Blanc et al. 2003; Bowers et al. 2003). The most recent WGD, the alpha WGD that is specific to the Brassicaceae family (Schranz and Mitchell-Olds 2006), accounts for about 2,500 pairs of duplicated genes in the *Arabidopsis* genome (Blanc et al. 2003; Bowers et al. 2003). Other kinds of gene duplication at small scales, such as tandem duplication, segmental duplication, and duplicative retroposition, have also continually enlarged the pool of duplicated genes.

Duplicated gene pairs may experience different fates. A likely outcome is that one copy is eventually lost or becomes a pseudogene. Some of the duplicated genes that are both preserved undergo regulatory neofunctionalization or subfunctionalization by gaining a new expression pattern or dividing the ancestral expression pattern between the duplicates, respectively (Force et al. 1999). Neofunctionalized genes often have experienced an accelerated rate of sequence evolution compared with paralogs; this will result in asymmetric nonsynonymous sequence evolution in duplicated gene pairs (e.g., Van de Peer et al. 2001; Byrne and Wolfe 2007; Liu et al. 2011; Owens et al. 2013). Several studies have explored the evolution of duplicate gene expression patterns in plants, and cases of regulatory neofunctionalization have been found by comparisons to the inferred ancestral (preduplication) state of expression (e.g., Duarte et al. 2006; Liu and Adams 2010; Liu et al. 2011).

Genomic imprinting is another way in which duplicated genes could diverge from each other by acquisition of imprinting by one copy (Walter and Paulsen 2003). Such a molecular mechanism, however, has received little attention to understand its relative contribution to the retention and divergence of duplicated genes, especially in plants. Genomic imprinting is when an allele is expressed or silenced depending on its parental origin, resulting in monoallelic expression. The phenomenon has been shown in mammals and in flowering plants. It is an epigenetic process involving DNA methylation and/or histone modifications (Berger and Chaudhury 2009; Kohler et al. 2012). A maternally imprinted gene has only the paternal allele showing expression (as a paternally expressed gene; peg hereafter). Similarly, a paternally imprinted gene has only the maternal allele showing expression (meg hereafter). In flowering plants, the imprinted expression is primarily within the endosperm, a tissue that facilitates the resource absorption from the maternal tissues to the embryo and that stores and provides nutrients to the embryo over the course of the seed development (Berger and Chaudhury 2009).

As of the beginning of 2011, there were only a few reported imprinted genes in flowering plants, including 11 in *Arabidopsis thaliana* and 6 in maize (reviewed in Berger and Chaudhury 2009; Raissig et al. 2011). Among those in *Arabidopsis*, a subset of the imprinted genes originated from gene duplication events, but only the evolutionary history of *MEDEA* has been studied in detail, revealing accelerated sequence rate evolution and expression divergence compared with its paralog *SWN* (Spillane et al. 2007; Miyake et al. 2009). During 2011, several genome-wide investigations of imprinting using RNA-seq approaches in *A. thaliana*, maize, and rice were published (Gehring et al. 2011; Hsieh et al. 2011; Luo et al. 2011; Waters et al. 2011; Wolff et al. 2011; Zhang et al. 2011). Three genome-wide studies in *Arabidopsis* together reported more than 300 imprinted genes, leading to an explosion of known imprinted genes in *Arabidopsis* (Gehring et al. 2011; Hsieh et al. 2011; Wolff et al. 2011).

In this study, we characterized sequence evolution and expression patterns of a large number of imprinted genes, which were formed during the evolution of the Brassicales, from a macroevolutionary and phylogenetic perspective in *A. thaliana*. This is the first large-scale study of sequence and expression evolution of duplicated imprinted genes in plants. We analyzed the sequence evolution of imprinted genes to determine whether there has been accelerated amino acid sequence evolution compared with their paralogs. We also compared expression patterns of imprinted genes with their paralogs and with orthologs in outgroup species to identify changes in expression profiles and infer if there has been regulatory neofunctionalization or subfunctionalization in the imprinted genes. Collectively, the data indicate that some imprinted genes show changes in expression profiles and accelerated sequence evolution after duplication, with some potentially having undergone neofunctionalization. Our results provide insights into the interplay between the origin of genetic imprinting and the retention of gene duplicates.

## Materials and Methods

### Phylogenetic Analyses

Sets of imprinted genes included 126 imprinted genes identified in Hsieh et al. (2011) and 65 imprinted genes identified in Wolff et al. (2011). To identify imprinted genes that arose from recent gene duplication events during the evolution of the Brassicales, phylogenetic analyses were performed on gene families containing imprinted genes. For each imprinted gene, coding region sequences (both nucleotide and amino acid) of family members from *A. thaliana*, *Carica papaya*, *Populus trichocarpa*, *Vitis vinifera*, *Ricinus communis*, *Manihot esculenta*, and *Zea mays* were obtained from PLAZA 2.0 (Proost et al. 2009). Amino acid sequences were aligned using MUSCLE with default settings (Edgar 2004), followed by manual refinement with BioEdit (Hall 1999). The aligned amino acid sequences were used as the reference to guide the nucleotide alignments using a customized Perl script. Maximum-likelihood trees based on the codon alignment were generated by RAxML v.7.0.3 and Garli v.1.0 where generalized time reversible (GTR) was used as the substitution model (Stamatakis 2006; Zwickl 2006). Bootstrapping with 100 replicates was applied to determine the statistical support for each clade. Tree topologies were compared with the expected species tree.

Imprinted genes were selected for subsequent analyses if they had recent paralogs that are specific to the Brassicales after the Brassicaceae family diverged from the Caricaceae family (supplementary fig. S1, Supplementary Material online). Orthologs of imprinted genes and their paralogs were identified according to the gene-tree topology and used for sequence rate analyses and expression assays. Imprinted genes that arose from the alpha WGD were identified with their paralogs according to Blanc et al. (2003) and Bowers et al. (2003). Imprinted genes that arose from recent tandem duplication were identified by the adjacent loci numbers and further confirmed according to those identified in Haberer et al. (2004).

### Detection of Asymmetric Sequence Evolution

To detect whether there has been an asymmetric sequence evolution in each duplicated pair, we followed the method described in Blanc and Wolfe (2004). For each imprinted gene, a triplet of amino acid sequences was constructed with the imprinted gene, its paralog, and an outgroup gene. Orthologs from *C. papaya*, *V. vinifera*, and *R. communis* were used as outgroup sequences one at a time. Those outgroup species were chosen because they do not have lineage-specific WGDs; thus, the orthologous genes in those species are generally single copy. These outgroup sequences were identified by phylogenetic analyses and then were confirmed by reciprocal best BLASTP hits, as in Hulsen et al. (2006) and Liu et al. (2011). Three individual rounds of analyses with different outgroup sequences were carried out. In each triplet, three sequences were aligned using MUSCLE with the default settings (Edgar 2004). All alignments were manually checked using BioEdit (Hall 1999). Gaps in outgroup sequences were compared with genomic sequences from GenBank to determine whether the gaps were real or errors caused by potential annotation problems. Then the sequence triplets were analyzed with two models of evolution: Unconstrained and clock-like. Model I assumed that all sequences are unconstrained to evolve at their unique rates, so all the branch lengths can be different. Model II assumed that the duplicates have the same rate, so the two branch lengths were set equal. The likelihood estimates were obtained using Codeml in PAML (Yang 2007). To test whether the two models are significantly different, a likelihood ratio test (LRT) was applied. Twice the difference of the likelihood estimates (*X* = 2 [ln 1 − ln 2]), where *X* indicates twice likelihood ratio, ln 1 indicates the likelihood estimate from Model I, and ln 2 indicates the likelihood estimate from Model II was compared against a χ^2^ distribution with one degree of freedom (df). A significant difference (*P* < 0.05) indicates that the duplicated pair has asymmetric sequence rate evolution. In a gene pair showing asymmetric evolution, whether the imprinted gene evolves faster or slower than its paralog was determined by comparison of the branch lengths estimated from Model I. For each gene pair, three separate tests were applied using different outgroup sequences as references, *Carica*, *Vitis*, and *Ricinus*, respectively. Thus, asymmetric evolution was determined according to the majority outcome (two or three out of three) from the three tests.

Imprinted genes tend to evolve faster compared with genes in *Arabidopsis* in general, when comparing sequence evolution rates between *A. thaliana* and *A. lyrata* (Wolff et al. 2011). We hypothesized that the imprinted genes and their paralogs would show asymmetric sequence rate evolution more often than other duplicated genes. We performed a χ^2^ test to analyze the imprinted subset of alpha WG duplicates compared with genome-wide alpha WG duplicates (from Blanc and Wolfe 2004).

### Microarray Analyses for Expression Breadth

*Arabidopsis thaliana* microarray data were obtained from the TAIR website (http://www.arabidopsis.org/, last accessed July 9, 2014). Data from 63 different organ types and developmental stages were included (Schmid et al. 2005) and were then normalized as in Liu et al. (2011). To compare the expression breadth of imprinted genes and their paralogs, we calculated two indices, expression width and organ specificity. Expression width is defined by the number of organ types and developmental stages in the total of 63 types that show a significant expression level of a gene. It is based on the presence or absence of expression in each organ type (Liu et al. 2011). A gene with broader expression would have a greater expression width. Expression organ specificity (τ) is calculated by the formula of Yang and Gaut (2011): $\tau =\sum_{j=1}^{n}\left(\frac{\left[1-S\left(i,j\right)/S\left(i,\max\right)\right]}{n-1}\right)$, where *n* = 63 is the number of organ types and *S* (*i,* max) is the highest log_2_ transformed expression value for gene *i* across the *n* organ types. A gene with expression limited to one or a few organ types or developmental stages would demonstrate a high organ specificity, whereas broadly expressed genes with similar expression levels in most organ types and developmental stages would have a low organ specificity value.

### Analyses of Asymmetric Sequence Evolution in Case Studies

For the gene pairs *MPC*/*PAB8*, *FWA* (*HOMEODOMAIN GLABROUS6* [*HDG6*])/*HDG7*, *HDG3*/*HDG2*, *HDG9*/*HDG10*, and *SUVH7*/*SUVH8*, additional selection analyses were performed. For each gene pair, sequences from *A. thaliana*, *C. papaya*, *V. vinifera*, *P. trichocarpa*, *R. communis*, and *M. esculenta* were aligned using MUSCLE with the default settings (Edgar 2004). Branchwise Ka/Ks ratios along the phylogenetic tree branches were estimated using a phylogeny-based free-ratio test using Codeml in PAML (Yang 2007). To test if the Ka/Ks ratio of imprinted genes and their paralogs evolved in an asymmetric fashion, two-ratio models and three-ratio models were compared. The first model assumes that the duplicates have one Ka/Ks ratio, whereas the orthologs have a different ratio. The second model assumes that the duplicates have different Ka/Ks ratios and thus the two genes evolved at different rates, with the third Ka/Ks ratio for the ortholog branch. An LRT was applied, where twice the different of likelihood values was calculated and compared against a χ^2^ distribution with df = 1. When the second model fits better than the first model with statistical support by an LRT, the evolutionary rate of the duplicated pair is determined to evolve in an asymmetric fashion.

A branch-site model (test 2) described in Yang et al. (2005) and Zhang et al. (2005) was applied to detect if specific codons in a certain gene sequence have experienced positive selection. Two hypotheses were compared, model A (null) and model A1 (alternative). The first model assumes the absence of positive selection, and the second model assumes the presence of positive selection. Then the LRT was applied to determine the significant difference between two models, where twice the difference of likelihood values was calculated and compared against a χ^2^ distribution with df = 1. Only codons that show posterior probability >0.95 from a Bayes Empirical Bayes analysis are considered to be positively selected sites.

### Plant Materials, Nucleic Acid Extraction, and RT-Polymerase Chain Reaction Expression Assays

Total RNA was extracted from *A. thaliana* (ecotype Col-0), *C. papaya* (cultivar Sun-Up), and *V. vinifera* (cultivar Pinot Noir), which were grown together in a greenhouse. For each species, five organ types were used for the RNA extraction: Roots, stems, leaves (rosette leaves in *Arabidopsis*), flowers, and seeds (whole siliques in *Arabidopsis*). Fresh plant materials were collected and frozen in liquid nitrogen. Total RNA of each sample was extracted as in Zhou et al. (2011). The quality and quantity of RNA was checked on agarose gels and by a spectrophotometer. DNase (Invitrogen) treatment was applied to remove the residual genomic DNA according to the manufacturer’s instructions. M-MLV reverse transcriptase (Invitrogen) was used to generate cDNA according to the manufacturer’s instructions and then polymerase chain reaction (PCR) was performed with cDNA templates. Gene-specific primers were designed to amplify 250–1,000 bp of the cDNA of targeted genes (supplementary table S1, Supplementary Material online). Primers were checked against the genome of each species, using BLASTn, to check for specificity to the target gene. For PCR analyses, the cycling programs were 94 °C for 3 min; 30–35 cycles of 94°C for 30 s, 55–58 °C for 30 s, 72 °C for 30 s, and 72 °C for 7 min. PCR products were checked on 1.2% agarose gels.

## Results

### A Large Number of Imprinted Genes Were Formed by Duplication During the Evolution of the Brassicales

To evaluate the duplication history of imprinted genes in *Arabidopsis*, we analyzed imprinted genes identified by two studies that used Illumina transcriptome sequencing in polymorphic F1 seeds of *A. thaliana* (Hsieh et al. 2011; Wolff et al. 2011). A total of 126 imprinted genes were identified by Hsieh et al. (2011), including 116 maternally expressed genes (megs) and 10 paternally expressed genes (pegs), and 65 imprinted genes were identified in Wolff et al. (2011) including 39 megs and 26 pegs. Together the two studies identified 183 imprinted and putatively imprinted genes including 149 megs and 34 pegs. We analyzed the set of imprinted genes in a phylogenetic context to identify those that arose by gene duplication during the evolution of the Brassicales (supplementary fig. S1, Supplementary Material online). We found that 125 out of the 183 imprinted genes (68%) originated from gene duplication events in the Brassicaceae lineage after it diverged from the Caricaceae lineage within the Brassicales (supplementary table S2, Supplementary Material online). Among the 125 imprinted genes, 54 genes were derived from the alpha WGD (gene pairs identified by Blanc et al. [2003] and Bowers et al. [2003]; gene pairs shown in supplementary table S3, Supplementary Material online), which are specific to the Brassicaceae family, and 44 genes by tandem duplication. Other kinds of gene duplication events also have contributed to the formation of the imprinted genes. Most of the paralogs of those imprinted genes are not reported as imprinted (Hsieh et al. 2011; Wolff et al. 2011), suggesting many imprinted genes might be specific to the Brassicaceae lineage, and that the acquisition of imprinting happened after those gene duplication events. However, neither Hsieh et al. (2011) nor Wolff et al. (2011) exhaustively identified imprinted genes in *A. thaliana,* and thus, it is possible that some of the paralogs of the imprinted genes, which were not identified as imprinted, are actually imprinted too.

### Asymmetric Sequence Evolution Is Common in Duplicated Pairs with Imprinted Genes

Duplicated genes sometimes show asymmetric sequence rate evolution, such that the amino acid sequence of one copy has evolved faster than the other copy. That phenomenon has been associated with functional diversification and neofunctionalization (Dermitzakis and Clark 2001; Blanc and Wolfe 2004; Kim and Yi 2006; Byrne and Wolfe 2007; Liu and Adams 2010). To test the hypothesis that duplicated genes with imprinting frequently show asymmetric sequence rate evolution compared with their paralogs, we used 46 alpha WGD gene pairs where one gene in the pair shows imprinting (Hsieh et al. 2011; Wolff et al. 2011). Gene pairs from the alpha WGD were used in this analysis because they were simultaneously duplicated and thus the results could be compared regardless of the age of the duplication. We used the method of Blanc and Wolfe (2004), which included an amino acid substitution rate analysis of the paralogs in *Arabidopsis* compared with an outgroup, to analyze sequence rate evolution in the pair after their formation by duplication.

**Fig. 1.—:**
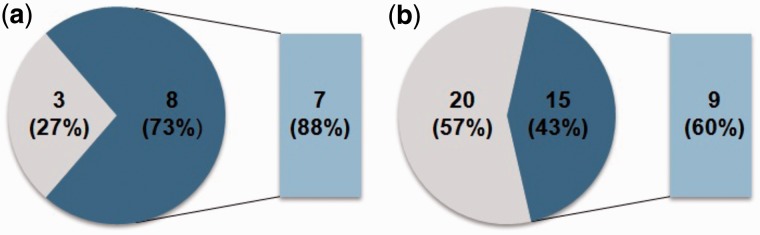
Asymmetric sequence rate evolution in duplicated gene pairs with imprinted genes. Pie charts indicating the number and percentage of asymmetrically evolving gene pairs (dark blue), the number and percentage of asymmetrically evolving gene pairs where the imprinted gene show faster sequence evolution than the paralog (light blue), and the number and percentage of symmetrically evolving gene pairs (gray). (*a*) Gene pairs with imprinted genes identified in Wolff et al. (2011). (*b*) Gene pairs with imprinted genes identified in Hsieh et al. (2011).

Of the imprinted genes identified by Hsieh et al. (2011), 15 out of 35 (43%) pairs of alpha WG duplicated genes show asymmetric protein sequence evolution, and within the 15 pairs, there are nine pairs (60%) where the imprinted genes evolved faster (fig. 1 and supplementary table S4, Supplementary Material online). In contrast, of the imprinted genes from Wolff et al. (2011), 8 out of 11 (73%) of alpha WG duplicated pairs have asymmetric protein sequence evolution, and 7 (88%) of those evolved faster than their paralogs (fig. 1 and supplementary table S4, Supplementary Material online). Thus, the imprinted genes often showed a faster rate of sequence evolution than their duplicated partners, especially among the genes identified in Wolff et al. (2011). They only analyzed genes with endosperm-specific expression in the seeds with the exclusion of genes having a broader expression pattern in seeds, suggesting that endosperm-specific imprinted genes more often showed a higher rate of sequence evolution than their duplicated partners. In contrast, Hsieh et al. (2011) applied microdissection to purify endosperm and then analyzed the endosperm transcriptome to find evidence for allelic biased expression and imprinting. Considering that the two research groups used different methodologies for identifying imprinted genes, we did not mix them and instead made them two sets of genes. AT1G17770 was identified as imprinted by both studies, and thus it is present in both data sets. Of the 15 imprinted genes that we found to evolve faster than their paralogs, eight have been shown to have functions, indicating that they are not pseudogenes, whereas the other seven have not been characterized. Those eight imprinted genes with known functions (in some cases, in other organ types and not the endosperm) include the histone H3K4 demethylase *JMJ15* (Yang et al. 2012), *CINV1* which plays roles in sugar metabolism and antioxidant defense (Xiang et al. 2011), *G6PD5* which is involved in oxidative stress responses (Wakao et al. 2008), *TAR1* which is involved in embryo patterning (Stepanova et al. 2008), *VEL2* which plays a role in repression of *FLC* gene family members during vernalization (Kim and Sung 2013), *PKR2* which is involved in activation of polycomb group target genes (Aichinger et al. 2009), *FWA* whose expression causes a late flowering phenotype (Kinoshita et al. 2004), and *HDG3* which functions in anther dehiscence (Li et al. 2007).

Blanc and Wolfe (2004) analyzed 833 duplicated gene pairs from the alpha WGD, using the same method to what we used, and found that 21% of them have evolved in an asymmetric pattern. That compares to 43% of the genes, we analyzed from Hsieh et al. (2011) and 73% of the genes we analyzed from Wolff et al. (2011). A χ^2^ test showed that the number of genes showing asymmetric rate evolution among the alpha WG duplicates with imprinted genes from Hsieh et al. (2011) and Wolff et al. (2011) is significantly different from the set of 833 alpha WGD genes analyzed by Blanc and Wolfe (2004) (χ*^2^*, *P* = 9.876e-4 and 7.266e-4). Thus, imprinted genes and their paralogs have a higher frequency of accelerated sequence evolution than gene pairs as a whole that were duplicated by the alpha WGD. In addition, among 267 alpha WGD pairs analyzed in Liu et al. (2011), only 16% showed an asymmetric sequence evolution pattern.

### Many Imprinted Genes Have Restricted Expression Patterns and Preferential Expression in Reproductive Organs

Duplicated gene pairs sometimes have contrasting expression patterns, such that one gene is broadly expressed in a wide range of organ and tissue types, whereas the other gene shows an expression pattern that is restricted to only a small number of organ types. We next tested the hypothesis that duplicated imprinted genes show a restricted expression pattern compared with their paralogs. We analyzed *Arabidopsis* ATH1 microarray data from 63 different organ types and developmental stages (Schmid et al. 2005). We used microarray data because data are available from a large number of organ types and developmental stages. Although imprinting is only found in seeds, imprinted genes are not necessarily only expressed in seeds. Many imprinted genes identified by Hsieh et al. (2011) and Wolff et al. (2011) have expression in vegetative and floral organ types as well. Thus, for both imprinted genes and their paralogs, we calculated the “expression width,” defined as how many organ types and developmental stages in which a gene has significant expression levels, and “expression organ specificity,” which indicates whether a gene has preferential expression in few organ types and developmental stages or broad expression in most organ types and developmental stages. We analyzed all gene pairs with imprinted genes that arose from the alpha WGD that were analyzed in the rate analyses in the previous section if microarray data were available for both copies. In some cases, microarray data were not available for one or both copies.

**Fig. 2.—:**
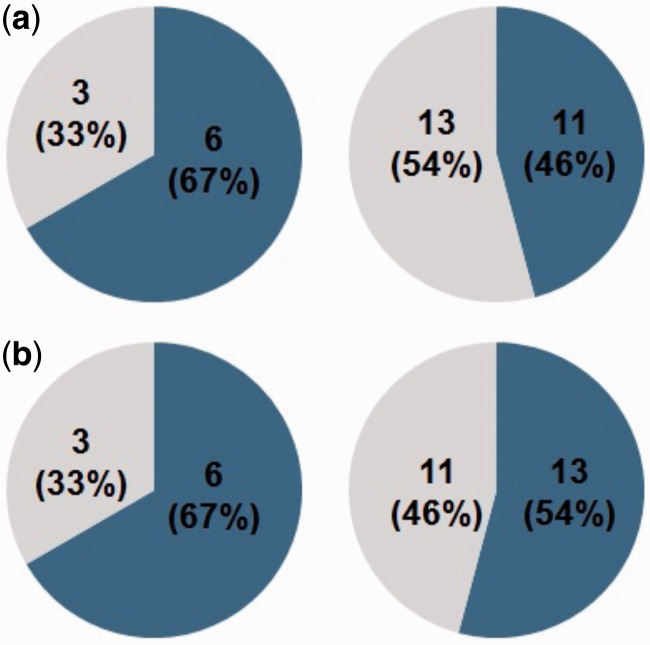
Many imprinted genes have a more restricted expression pattern compared with their paralogs. (*a*) Expression width, based on microarray data. Dark blue: Number and percentage of imprinted genes with a smaller expression width. Gray: Number and percentage of imprinted genes with a larger expression width. (*b*) Organ specificity of expression, based on microarray data. Dark blue: Number and percentage of imprinted genes with a higher organ specificity of expression. Gray: Imprinted genes with a lower organ specificity of expression. In panels (*a*) and (*b*), gene pairs with imprinted genes identified in Wolff et al. (2011) are on the left, and gene pairs with imprinted genes identified in Hsieh et al. (2011) are on the right.

For imprinted genes from Hsieh et al. (2011), 11 out of 24 (46%) of the imprinted genes have a smaller width compared with their paralogs, which means that they are expressed in fewer organ types than their paralogs (fig. 2 and supplementary table S5, Supplementary Material online). Calculating the expression specificity in different organ types, 13 out of 24 imprinted genes (54%) have higher expression organ specificity than the paralogs; nine pairs overlapped in the two analyses. A similar trend is more apparent with imprinted genes from Wolff et al. (2011): Six out of nine imprinted genes have limited expression, and six out of nine imprinted genes have higher organ specificity of expression (fig. 2 and supplementary table S5, Supplementary Material online). The imprinted genes with limited expression patterns also have higher expression levels in reproductive organs. Overall, the reproductive organ-specific expression is a relatively common feature of imprinted genes, which contrasts to the broadly expressed paralogs.

When comparing the results from the expression analysis and asymmetric sequence rate analysis, we observed that many imprinted genes that evolved faster than the paralogs also show a restricted expression pattern. In Hsieh et al. (2011), nine imprinted genes evolved faster than their paralogs, and five of them have at least one index indicating restricted expression, whereas there is no expression data for two imprinted genes. In Wolff et al. (2011), seven imprinted genes evolved faster than their paralogs, and five of them have at least one index indicating restricted expression, whereas two imprinted genes have no data. In total, 15 imprinted genes (AT1G17770 identified by both studies) evolved faster than their paralogs, ten of them have a restricted expression profile, two do not, and expression data are not available for three others.

### Expression Patterns of Imprinted Genes Changed after Gene Duplication to Become Restricted to Reproductive Organs

We tested the hypothesis that the imprinted genes with reproductive organ-restricted expression might result from the loss of expression in vegetative organs by investigating the expression pattern of orthologs from *Carica* and *Vitis* to infer the preduplication ancestral expression pattern. We examined six duplicated pairs, which have imprinted genes with reproductive organ restricted expression patterns and paralogous partners with broad or nonreproductive organ restricted expression patterns.

**Fig. 3.—:**
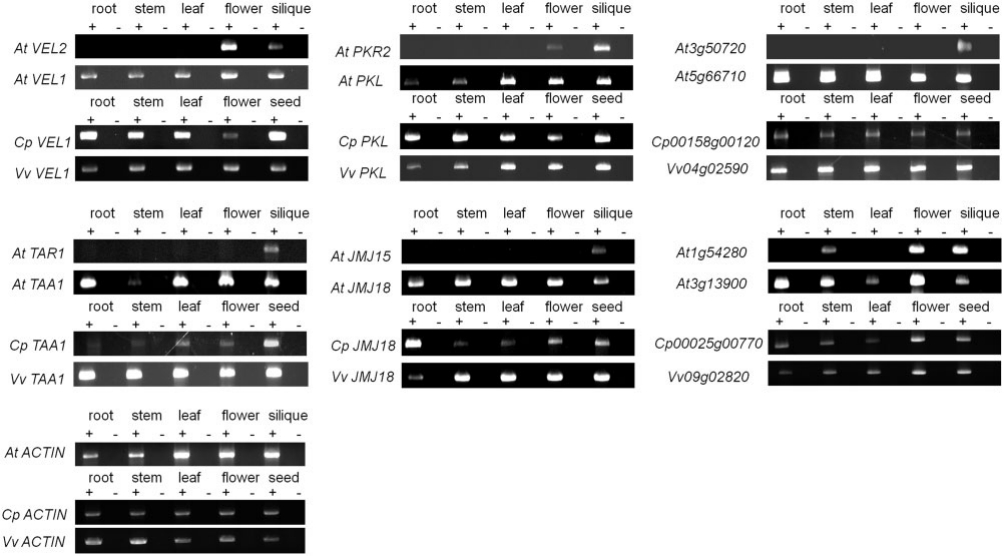
RT-PCR results of imprinted genes, their paralogs, and orthologs from outgroup species. RT-PCR expression assays were performed using five organ types: Root, stem, leaf, flower, and silique (*Arabidopsis*)/seed (*Carica* and *Vitis*), listed above the corresponding columns. Plus signs indicate the presence of reverse transcriptase in the reaction, and minus signs indicate the absence of reverse transcriptase as negative controls. Gene pairs in *Arabidopsis thaliana* (*At*), and their orthologs from outgroup species, *Carica papaya* (*Cp*), and *Vitis vinifera* (*Vv*) are listed beside the corresponding panels. *ACTIN* in each species was used as a positive control for RT-PCR, and the results are shown in the bottom right graph.

Five of the six imprinted genes are only expressed in flowers and/or siliques of *Arabidopsis* (fig. 3): The maternally expressed genes *JMJ15* (AT2G34880, a histone demethylase) and *VEL2* (AT2G18880, vernalization-related protein 5), along with the paternally expressed genes *TAR1* (AT1G23320, tryptophan aminotransferase-related protein), *PKR2* (AT4G31900, pickle related-protein 2), and AT3G50720 (a protein kinase). In contrast, the alpha WGD paralogs all have broad expression in both vegetative and reproductive organs (fig. 3). The *Carica* and *Vitis* orthologs of each of the six gene pairs from *Arabidopsis* are broadly expressed (fig. 3), indicating that the preduplication expression pattern is likely to be a broad expression pattern. Thus, the imprinted genes in *Arabidopsis* likely lost expression in vegetative organs. This is consistent with the genes becoming specialized in reproductive organs.

### *Expression, Sequence, and Structural Evolution of* MPC*, *FWA*, and *HDG3

We studied sequence evolution and expression patterns of three imprinted genes in more detail. *MPC*, *FWA* (or *HDG6*), and *HDG3* were among the initial imprinted genes to be discovered in *A. thaliana* before the high-throughput sequencing studies in 2011. All three genes were derived from the Brassicaceae-specific alpha WGD (Blanc et al. 2003; Bowers et al. 2003; supplementary fig. S2, Supplementary Material online). The *PAB8* paralog of *MPC* encodes a poly(A) binding protein that plays a role in mRNA stability and translation, whereas maternally expressed *MPC* encodes a truncated protein that only has one-fourth the length of regular PAB proteins at the C-terminal domain (Tiwari et al. 2008). *FWA* has maternal expression in the female gametophyte and silique (Nakamura et al. 2006), and its ectopic expression in vegetative tissue causes a late flowering phenotype (Kinoshita et al. 2004); in contrast, its paralog *HDG7* functions in vegetative meristems (Nakamura et al. 2006). *HDG3* is a paternally expressed gene (Gehring et al. 2009) that may function in the regulation of anther dehiscence (Li et al. 2007). The function of its paralog *HDG2* is related to epidermal development such as trichome and stoma differentiation (Marks et al. 2009; Peterson et al. 2013), as well as for the identity of petals and stamens (Kamata et al. 2013).

**Fig. 4.—:**
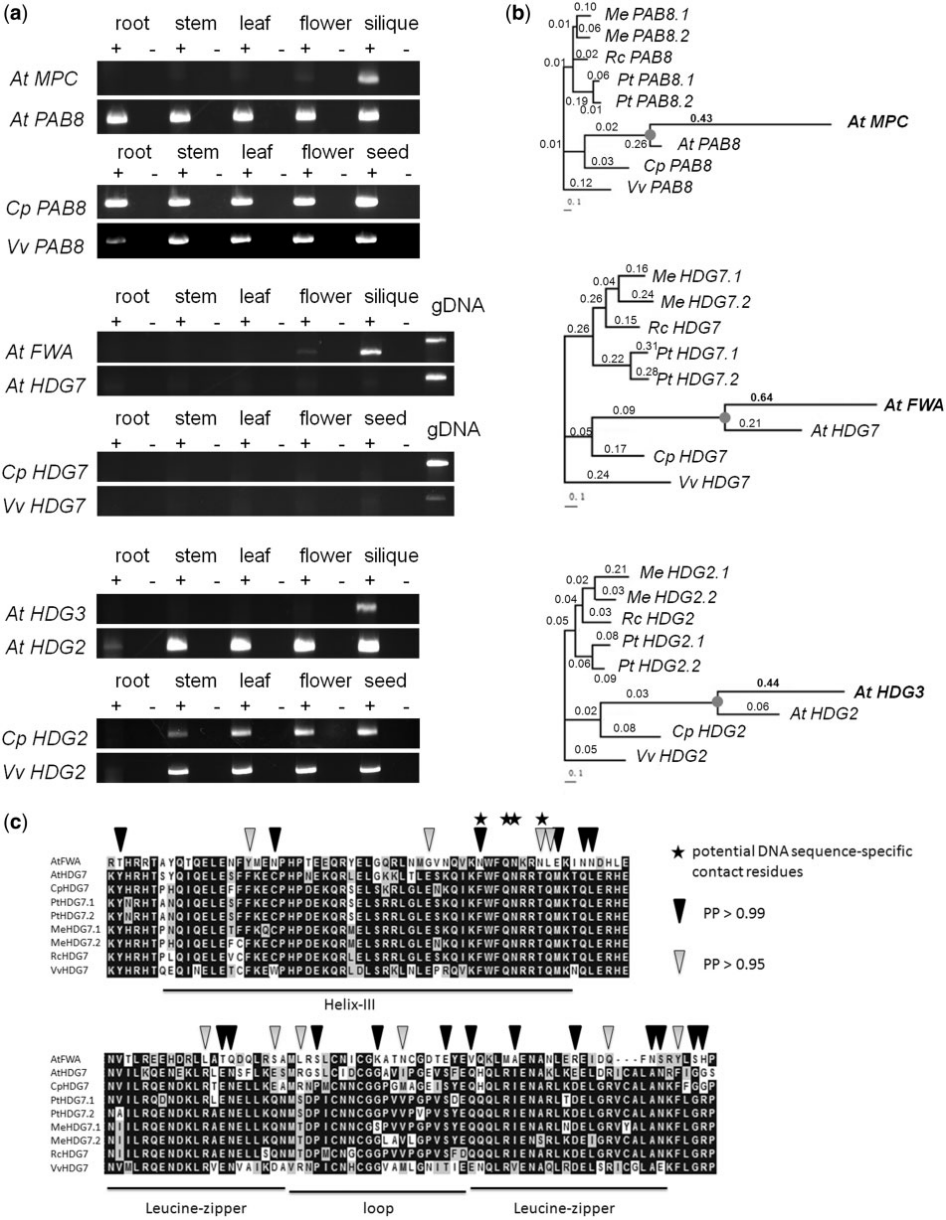
Analyses of *MPC*, *FWA*, and *HDG3*. (*a*) RT-PCR expression assays were performed using five organ types: Root, stem, leaf, flower, and silique (*Arabidopsis*)/seed (*Carica* and *Vitis*), listed above the corresponding columns. Plus signs indicate the presence of reverse transcriptase in the reaction, and minus signs indicate the absence of reverse transcriptase as negative controls gDNA, genomic DNA. Gene pairs in *Arabidopsis thaliana* (*At*), and their orthologs of *MPC*/*PAB8*, *FWA*/*HDG7*, and *HDG3*/*HDG2* from outgroup species, *Carica papaya* (*Cp*) and *Vitis vinifera *(*Vv*) are listed beside the corresponding panels. (*b*) Sequence rate analyses. Phylogenetic trees of each gene have sequences from *A. thaliana *(*At*), *C. papaya* (*Cp*), *Populus trichocarpa *(*Pt*), *Ricinus communis *(*Rc*), *Manihot esculenta *(*Me*), and *V. vinifera *(*Vv*). Trees are unrooted. Branch lengths were generated by Codeml in PAML, and the scale bar indicates nucleotide substitutions per codon. Branch-wise Ka/Ks ratios are indicated above the branches. Imprinted genes and their larger Ka/Ks ratios are shown in bold. Circles indicate the gene duplication events that gave rise to the imprinted genes and their paralogs. (*c*) Site-specific selection analysis on FWA HD-ZIP domain to detect positively selected amino acid sites. An amino acid alignment of the HD-ZIP domain in FWA and HDG7 in *A. thaliana* (*At*), and orthologous genes from *C. papaya* (*Cp*), *P. trichocarpa* (*Pt*), *R. communis* (*Rc*), *M. esculenta* (*Me*), and *V. vinifera* (*Vv*) showing the amino acid substitution in FWA. Residues with more than 50% shared identity are black shaded, and similar amino acids are gray shaded. Motifs are labeled under the bar below the alignment. Stars indicate potential DNA sequence-specific contact residues that are conserved in all *At*HDG family members but FWA. Positively selected sites on FWA were marked by triangles, where black triangles indicate posterior probabilities (PP) greater than 0.99 and gray triangles indicate PPs greater than 0.95.

*MPC* and *HDG3* have expression only in siliques (fig. 4), whereas their paralogs *PAB8* and *HDG2* are broadly expressed. To test the hypothesis that the expression of the imprinted genes became more restricted after gene duplication, we reconstructed its ancestral expression profile by assaying the expression pattern of orthologs in *Carica *and *Vitis*. The *MPC/PAB8* and *HDG3/HDG2* orthologs in *Carica* and *Vitis* were both broadly expressed. That suggested that the ancestral expression state was broad expression, and that *MPC* and *HDG3* acquired a silique-specific expression pattern after duplication. *FWA* is expressed in flowers and seeds (fig. 4*a*). In contrast, *HDG7* is expressed only in young vegetative tissues, such as root and stem tips (Nakamura et al. 2006), and probably because of its low expression level in limited tissue types, the expression was not detected by RT-PCR (fig. 4*a*). The *FWA/HDG7* orthologs in *Carica *and *Vitis *had a similar expression pattern to *HDG7* (fig. 4*a*) suggesting that *HDG7* has the ancestral state of expression and the expression pattern of *FWA* in siliques was derived after gene duplication.

We further characterized sequence rate evolution in *MPC* and *PAB8*, *FWA* and *HDG7*, and *HDG3* and *HDG2* using a free-ratio analysis in PAML to calculate the Ka/Ks ratio of imprinted genes and their paralogs. We found that *MPC*, *FWA*, and *HDG3* all experienced accelerated sequence rate evolution (i.e., higher Ka/Ks ratio) compared with their paralogs (fig. 4*b*). The Ka/Ks ratio of *MPC* is approximately twice that of *PAB8*, and the Ka/Ks ratio of *FWA* is approximately three times that of *HDG7*. *HDG3* has a Ka/Ks ratio approximately seven times that of *HDG2*. Branch site-specific positive selection analysis indicated that the HD-ZIP domain of FWA has several amino acid sites that show evidence for positive selection (fig. 4*c*). It is noteworthy that of four potential DNA sequence-specific contact residues of *Arabidopsis *HDG proteins that are conserved across the HDG proteins (Nakamura et al. 2006), two of them are not conserved in FWA and those sites show positive selection (fig. 4*c*). Together, the detection of positive selection and the evolution of a new expression profile in *FWA* suggest neofunctionalization of *FWA*.

### Oppositely Imprinted Gene Duplicates with Rapid Sequence Evolution

**Fig. 5.—:**
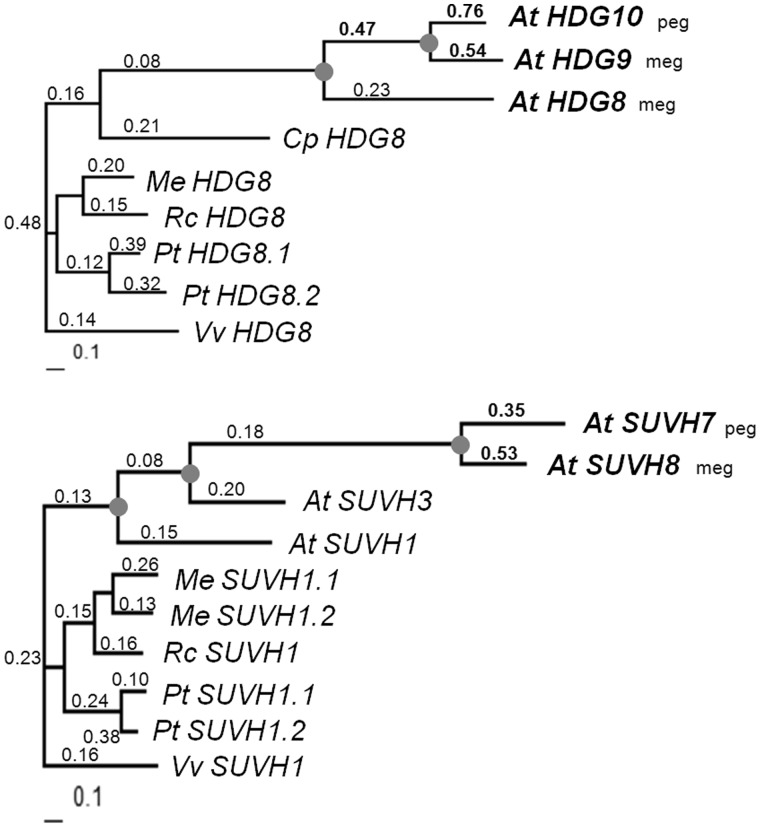
Sequence rate analyses of *HDG9*/*HDG10* and *SUVH7*/*SUVH8*. Phylogenetic trees of each gene have sequences from *Arabidopsis thaliana *(*At*), *Carica papaya* (*Cp*), *Populus trichocarpa *(*Pt*), *Ricinus communis *(*Rc*), *Manihot esculenta *(*Me*), and *Vitis vinifera *(*Vv*). Trees are unrooted. Branch lengths were generated by Codeml in PAML, and the scale bar indicates nucleotide substitutions per codon. Branch-wise Ka/Ks ratios are indicated above the branches. Imprinted genes are shown in bold. The suffix of *Arabidopsis* genes indicates the imprinting status: meg versus peg. High Ka/Ks ratios of imprinted genes or their inferred precursor are shown also in bold. Circles indicate the gene duplication events that gave rise to the imprinted genes and their paralogs.

Two paralogous gene pairs that are both imprinted have imprinting on opposite alleles. They are *HDG9* and *HDG10* that are members of the HD-ZIP IV gene family, along with *SUVH7* and *SUVH8* that encode SET domain proteins. Described as imprinted by Gehring et al. (2009), *HDG9* is a meg with exclusively maternal expression, and its alpha WG duplicate, *HDG8* is a primarily maternally expressed gene. In contrast, *HDG10*, which was derived from *HDG9*, was identified by Wolff et al. (2011) as a peg. A second case is *SU(VAR)3-9 HOMOLOG 7* (*SUVH7*) which is a peg, and *SUVH8* which is a meg, both of which were identified in Hsieh et al. (2011). They have an alpha WG duplicate *SUVH3*, as well as another duplicate *SUVH1* both of which to appear to be nonimprinted (Hsieh et al. 2011). We characterized the sequence rate evolution of these imprinted duplicated gene pairs in comparison with their paralogs (fig. 5). We found that both *HDG9* and *HDG10* evolved significantly faster than *HDG8* and most orthologs from outgroup species. We found the Ka/Ks ratios of *HDG9* and *HDG10* are approximately 2.5 and 3.5 times higher than *HDG8*. *HDG10* evolved slightly faster than *HDG9*, but the difference is not statistically significant. Similarly, both *SUVH7* and *SUVH8* have a higher Ka/Ks ratio than *SUVH3* or *SUVH1*, indicating relaxation of purifying selection on the two imprinted genes. Considering that the branch leading to the *HDG9*/*HDG10* has a significantly higher Ka/Ks ratio than *HDG8* and the preorthologous branch, we could infer that the ancestral gene of *HDG9*/*HDG10* was already evolving more rapidly than *HDG8* (fig. 5). However, this is not seen for the most recent common ancestral gene of *SUVH7*/*SUVH8*, which shows a Ka/Ks ratio similar to *SUVH3* and significantly lower than either of its descendants, *SUVH7* and *SUVH8*, indicating *SUVH7* and *SUVH8* started to accelerate in sequence evolution independently after diverging from each other (fig. 5). One possible explanation might be that the precursor of *HDG9*/*HDG10* was also a meg just like *HDG8* and evolved at a rapid rate. For *SUVH7* and *SUVH8*, their precursor was likely to be a nonimprinted gene as is *SUVH3*. Then, they were recruited into genomic imprinting independently after their split, along with both sequences evolving rapidly.

## Discussion

### Imprinted Genes with Endosperm-Preferential Expression in Seeds Frequently Show Evidence of Possible Neofunctionalization After Gene Duplication

After their formation, duplicated gene pairs may have different fates. Those include functional diversification and neofunctionalization, regulatory neofunctionalization, subfunctionalization, and other kinds of changes in expression patterns, among other fates. Genes that experienced neofunctionalization can show a novel expression pattern, rapid amino acid substitution rates, and sometimes evidence for positive selection (e.g., Blanc and Wolfe 2004; Byrne and Wolfe 2007; Liu and Adams 2010). In this study, we tested the hypotheses that duplicated imprinted genes show accelerated sequence evolution compared with their paralogs, that they show expression profiles restricted to flowers and seeds in contrast to their broadly expressed paralogs, and that the flower and seed specific expression was derived after gene duplication. We found that after gene duplication, many imprinted genes have evolved changes in expression profile to become restricted to flowers and seeds. Of the genes with endosperm-specific expression in the seed from Wolff et al. (2011), 67% showed expression limited to reproductive organs. Based on RT-PCR assays of orthologs in outgroup species, the restricted expression pattern of the imprinted genes appears to be a derived state after gene duplication. In addition, many of the duplicated gene pairs (73% of genes examined from Wolff et al. [2011] and 43% of the genes examined from Hsieh et al. [2011]) show asymmetric sequence rate evolution, which is a significantly higher fraction than alpha WG duplicated pairs in general (21% in Blanc and Wolfe [2004] and 16% in Liu et al. [2011]). Together, our findings from the expression and sequence rate analyses suggest that many of the imprinted genes may have undergone neofunctionalization. Although some imprinted duplicated genes did not show those characteristics compared with their paralogs, one possible explanation could be that they were recently recruited to genomic imprinting and there has been insufficient time for neofunctionalization to occur.

Although the general trend is similar in all genes analyzed, the features of imprinted genes in Hsieh et al. (2011) are different from those in Wolff et al. (2011) to some extent. The majority of the imprinted genes in Wolff et al. (2011) have a restricted expression pattern and an accelerated sequence evolution rate compared with their paralogs; however, although this trend is found in imprinted genes in Hsieh et al. (2011), it is less obvious than in the genes from Wolff et al. (2011). This is probably because of the differences between the criteria in assaying and filtering for imprinted genes. When sequencing and analyzing the endosperm transcriptome from small seeds to identify imprinted genes, it is difficult to avoid contamination from maternal tissues, such as the seed coat or nucellus. The potential contamination would bring in more maternal transcripts and lead to an artifact of more expression of maternal alleles, resulting in false positives in meg identification or failure in peg identification. To minimize maternal tissue contamination and increase the confidence of imprinted gene identification, different approaches were carried out in the two studies. In Hsieh et al. (2011), laser capture microdissection was applied to isolate the endosperm. As microdissection technically eliminated the maternal tissue, the transcripts were considered to be from pure endosperm. In Wolff et al. (2011), in contrast, the transcriptome from the whole seeds was sequenced. However, only genes with preferential endosperm expression, but not expression in the seed coat or other parts of the seed according to microarray data, were subsequently analyzed. The different criteria to avoid maternal contamination may lead to the different nature of the two sets of genes: The genes in Wolff et al. (2011) are strictly endosperm-specifically expressed in seeds, and thus they are selected to have limited expression in seeds, but not necessarily other organ types, at the first experimental step in the original study. In contrast, imprinted genes identified in Hsieh et al. (2011) were not preselected for expression width in seeds. After the publications of Hsieh et al. (2011) and Wolff et al. (2011), Gehring et al. (2011) reported 208 imprinted or partially imprinted genes in the endosperm, including 165 megs and 43 pegs. Similar to Hsieh et al. (2011), Gehring et al. (2011) applied dissection of seeds to obtain purified transcriptome from the endosperm, but they used different methods of data analysis. We decided to use Hsieh et al. (2011) and Wolff et al. (2011) because they represented different approaches to obtain the transcriptome of endosperm but applied similar statistical analyses and criteria for identifying imprinted genes. Also Hsieh et al. (2011) and Wolff et al. (2011) did more follow-up RT-PCR and sequencing verification of the newly identified imprinted genes.

### *Neofunctionalization of *MPC*,* FWA, *and *HDG3

Imprinted *MPC* and nonimprinted *PAB8* are a Brassicaceae-specific alpha WGD pair. However, unlike most other genes duplicated by the alpha WGD, *MPC* is just one-fourth of the total length of *PAB8*, aligning only at the 3′-end (in the C-terminus of the corresponding protein). In addition to having a new limited expression pattern (fig. 4*a*), *MPC* has a different function as a truncated protein (Tiwari et al. 2008). PAB proteins are mRNA polyA binding proteins. They bind the polyA tails of mRNAs through N-terminal RNA recognition motifs and interact with other proteins through the C-terminus, affecting mRNA stability and regulating translation. The C-terminus is very conserved and is recognized by CTC-interacting domain (CID) proteins carrying a PAM2 domain. MPC could be regarded as a pure PAB C-terminus. It might bind to the PAM2 domain and block the interaction with a complete PAB protein. However, MPC has lost the mRNA binding domain, as a result down-regulating the activities of other PAM2 domain containing CID proteins (Tiwari et al. 2008). Thus, in addition to accelerated sequence rate evolution (fig. 4*b*) and a new restricted expression pattern in reproductive organs (fig. 4*a*), MPC has acquired a new function through the loss of N-terminus (5′-end on corresponding mRNA) mRNA binding domains.

Both *FWA* (*HDG6*) and *HDG7* are HD-ZIP class IV proteins, which are characterized by the homeodomain helix III followed by the leucine zipper-loop-zipper motif (Nakamura et al. 2006). They are transcription factors but regulate transcription in different manners. *HDG7* is expressed in primordial parts of vegetative organs and functions in the epidermal layer of the apical meristem. HDG7 was observed binding epidermal-like box sequences and likely regulates the epidermal layer-specific expression (Nakamura et al. 2006). However, *FWA* has a novel expression pattern in reproductive organs that is different from *HDG7* and the orthologs from outgroup species (fig. 4*a*). *FWA* expression is female gametophyte- and endosperm specific, and the epigenetic *FWA* mutant with ectopic *FWA* expression has a late-flowering phenotype (Kinoshita et al. 2004). Although its epigenetic regulation has been extensively studied, the function of *FWA* in seeds is not yet known. The accelerated sequence evolution and site-specific positive selection in *FWA* (fig. 4*b* and *c*) may have been involved in *FWA* gaining a new function that is different from *HDG7*.

*HDG3* and *HDG2* are another paralogous pair in the *HDG* family, and their most closely related paralogs in *HDG* family are *ATML1* and *PDF2* (Peterson et al. 2013). Similar to *HDG7* and several other *HDG* genes, *HDG2* along with *ATML1* and *PDF2* has meristem-enriched expression (Nakamura et al. 2006). Partially redundant with *ATML1* and *PDF2*, *HDG2* functions in regulating the development of epidermis and the fates of epidermal cells. For example, *HDG2* affects trichome cell wall formation and wax deposition, as well as the differentiation and development of stomata (Marks et al. 2009; Peterson et al. 2013). Overexpression of *HDG2* induced multiple epidermal layers and ectopic stomata in mesophyll (Peterson et al. 2013). *HDG2* was also reported to show expression in floral primordial tissue, and it is important in determining the identity of petals and stamens (Nakamura et al. 2006; Kamata et al. 2013). Overall, *HDG2* plays roles functioning in primordial or epidermal development throughout all life stages in plants. In contrast, HDG3 does not have the epidermal-like box interacting motif (Nakamura et al. 2006), so it probably no longer functions in epidermal tissues. HDG3 was shown to function in anther dehiscence (Li et al. 2007), whereas its possible role in seed development is not clear. The loss of expression in vegetative organs, along with the rapid sequence evolution, is consistent with the possibility that *HDG3* has gained a novel function in reproductive organs and it is not redundant with *HDG2*.

### Multiple Recent Origins of Imprinting through Gene Duplication in the Brassicales

Our finding that 125 imprinted genes originated by duplication during the evolution of the Brassicales, which is 68% of imprinted genes that were identified by two large-scale imprinted gene identification studies (Hsieh et al. 2011; Wolff et al. 2011), suggests that imprinting might be related to duplicate gene retention in plants. In only a few cases was the paralog identified as imprinted in the studies of Hsieh et al. (2011), Wolff et al. (2011), or Gehring et al. (2011). Thus, imprinting may have arisen after gene duplication. However, for some genes, there were not enough RNA-seq reads with informative single nucleotide polymorphism (SNP) sites to be able to reliably assess the imprinting status, and for other genes, there were no SNPs between the alleles. Thus, the lack of identification of the paralogs as imprinted does not necessarily mean that most of them are not imprinted. Further studies with deeper Illumina sequencing or RT-PCRs assays of individual genes, as well as using genotypes with SNPs between the maternal and paternal alleles for some genes, will be necessary for determining whether or not all of the paralogs are imprinted. However, it is likely that many of the paralogs of the 125 imprinted genes are not imprinted. One example is the nonimprinted *PAB8*, which is the paralog of imprinted *MPC* (Tiwari et al. 2008). There is also the possibility of loss of imprinting in one gene after duplication, and this possibility could be evaluated by assaying the imprinting status in orthologous genes from outgroup species to infer if the ancestral, preduplication state was imprinted.

Our findings suggest that genomic imprinting can be a factor in duplicate gene divergence, so as to induce the diversification and retention of duplicates. The incidence of imprinted genes is related to the activities of transposable elements (TEs), which may be random events, not directional (Wolff et al. 2011). TEs are active sites for epigenetic regulation, which also tend to cause changes in expression profiles of nearby imprinted genes. That may be a factor causing the new restricted expression profiles of many of the imprinted genes compared with their paralogs. The restricted expression profile, along with accelerated amino acid sequence evolution, may lead to functional specialization of some of the imprinted genes in reproductive organs, as seen in *FWA* and *HDG3*. Functional diversification and neofunctionalization could contribute to duplicate gene retention. Another possible explanation for the correlation between genomic imprinting and retention of gene duplicates is that imprinting may be a way of adjusting the expression levels of duplicated genes when expressed in the endosperm. This is especially applicable to those duplicated genes derived from small-scale gene duplication events. After tandem gene duplication or other kinds of small-scale duplication, the dosage of gene products may increase, whereas acquisition of imprinting can result in the compensation for the increased dosage (Gehring et al. 2011; Yoshida and Kawabe 2013).

## Supplementary Material

Supplementary figures S1 and S2 and tables S1–S5 are available at *Genome Biology and Evolution* online (http://www.gbe.oxfordjournals.org/).

Supplementary Data
