# Supplementary material for: Frequent Changes in Expression Profile and Accelerated Sequence Evolution of Duplicated Imprinted Genes in Arabidopsis
Source: Genome Biol Evol. 2014 Jul 2;6(7):1830–42. doi: 10.1093/gbe/evu144 (PMC4122942; doi:10.1093/gbe/evu144)
Supplement: Supplementary Data [file supp_6_7_1830__index.html]

Supplementary Data 

# Frequent Changes in Expression Profile and Accelerated Sequence Evolution of Duplicated Imprinted Genes in *Arabidopsis*

## Supplementary Data

file

**Files in this Data Supplement:**

- Supplementary Data - pdf file
- Supplementary Data - xlsx file
