# Supplementary material for: Frequent Changes in Expression Profile and Accelerated Sequence Evolution of Duplicated Imprinted Genes in Arabidopsis
Source: Genome Biol Evol. 2014 Jul 2;6(7):1830–42. doi: 10.1093/gbe/evu144 (PMC4122942; doi:10.1093/gbe/evu144)
Supplement: Supplementary Data [file supp_evu144_evu144-Figure_S1_and_S2.pdf]

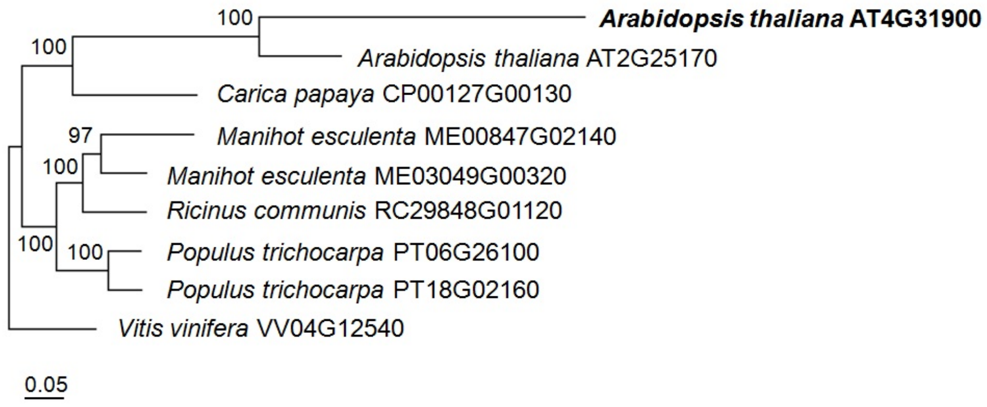

Supplementary Fig. S1. A phylogenetic tree showing genes formed by duplication after the Brassicaceae diverged from the Caricaceae. Bootstrap support values are labeled above the branches. The gene in bold is imprinted.

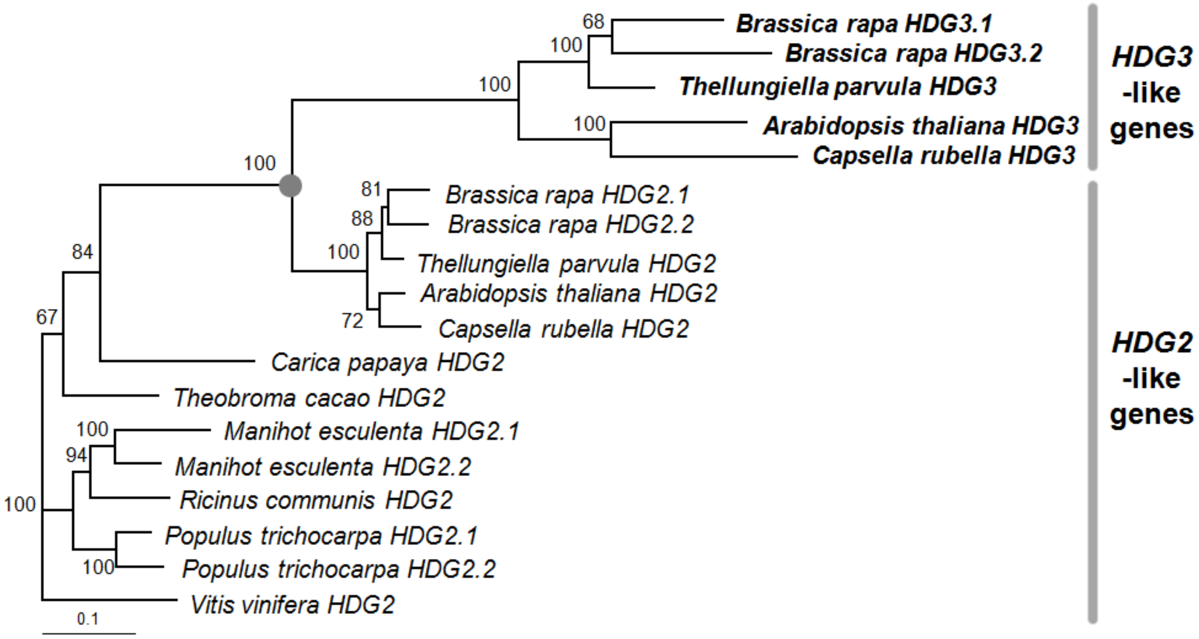

Fig. S2. Phylogenetic tree of *HDG3*/*HDG2* in rosids. *HDG3* and *HDG2* are a duplicated gene pair in the HD-ZIP IV family. Although Nakamura et al. (2006) reported their formation by duplication as being before the divergence between gymnosperms and angiosperms, our phylogenetic tree analysis indicates it occurred at the base of the Brassicaceae family, which is supported by two structural studies of genes derived from the alpha whole genome duplication (Blanc et al. 2003; Bowers et al. 2003). A maximum likelihood tree based on the codon alignment was generated by RAxML v.7.0.3 where GTR was used as the substitution model (Stamatakis 2006). Bootstrapping with 200 replicates was applied to determine the statistical support for each clade. The circle indicates the gene duplication event that gave rise to *HDG3*. Sequences include the following *HDG3*/*HGD2* sequences: *Arabidopsis thaliana*, *Carica papaya*, *Populus trichocarpa*, *Ricinus communis*, *Manihot esculenta*, *Vitis vinifera* and *Theobroma cacao* from PLAZA 2.5 (http://bioinformatics.psb.ugent.be/plaza/), *Capsella rubella* from Phytozome v8.0 (http://www.phytozome.org/), *Thellungiella parvula* from the *Thellungiella* genomics website (http://thellungiella.org/), and *Brassica rapa* from the BRAD *Brassica* database (http://brassicadb.org/brad/).
